# Mechanical Properties and Biological Behavior of 3D Matrices Produced by Electrospinning from Protein-Enriched Polyurethane

**DOI:** 10.1155/2018/1380606

**Published:** 2018-06-26

**Authors:** Vera S. Chernonosova, Alexander A. Gostev, Yun Gao, Yuriy A. Chesalov, Alexey V. Shutov, Evgeniy A. Pokushalov, Andrey A. Karpenko, Pavel P. Laktionov

**Affiliations:** ^1^Institute of Chemical Biology and Fundamental Medicine, Siberian Branch, Russian Academy of Sciences, Novosibirsk 630090, Russia; ^2^Meshalkin National Medical Research Center, Ministry of Health of the Russian Federation, Novosibirsk 630055, Russia; ^3^Novosibirsk State University, Novosibirsk 630090, Russia; ^4^Boreskov Institute of Catalysis, Siberian Branch, Russian Academy of Sciences, Novosibirsk, 630090, Russia; ^5^Lavrentyev Institute of Hydrodynamics, Siberian Branch, Russian Academy of Sciences, Novosibirsk, 630090, Russia

## Abstract

Properties of matrices manufactured by electrospinning from solutions of polyurethane Tecoflex EG-80A with gelatin in 1,1,1,3,3,3-hexafluoroisopropanol were studied. The concentration of gelatin added to the electrospinning solution was shown to influence the mechanical properties of matrices: the dependence of matrix tensile strength on protein concentration is described by a bell-shaped curve and an increase in gelatin concentration added to the elasticity of the samples. SEM, FTIR spectroscopy, and mechanical testing demonstrate that incubation of matrices in phosphate buffer changes the structure of the fibers and alters the polyurethane-gelatin interactions, increasing matrix durability. The ability of the matrices to maintain adhesion and proliferation of human endothelial cells was studied. The results suggest that matrices made of 3% polyurethane solution with 15% gelatin (wt/wt) and treated with glutaraldehyde are the optimal variant for cultivation of endothelial cells.

## 1. Introduction

The micro- and nanofiber matrices produced by electrospinning using solutions of synthetic or natural polymers as well as their blends can be made sufficiently durable depending on the used polymeric composition. Electrospun matrices can simulate the structure of extracellular matrix, possess good biocompatibility, ability to be colonized by cells, and integrate with the adjacent tissues. As such, they are widely used in engineering of soft and hard tissues (nerves, blood vessels, skin, cartilage, bone, etc.) [1, 2]. Such matrices are frequently made of polyurethanes (PUs)—polymers comprised of alternating hard (diisocyanate) and soft (dyol) segments. Depending on the chemical nature of these segments, PUs have different elasticity, strength, biocompatibility, and stability in the biological media [3–6]. PUs initially emerged as thermoplastic polymers widely used for manufacture of biological 3D matrices by electrospinning [7]. PU-based 3D matrices have been previously used in the production of various tools for cardiovascular surgery [8–10], implanted and external devices [11–13]. During electrospinning the fiber is formed from a polymeric solution or a mixture of polymers, allowing this method to produce fibers enriched by proteins. Addition of extracellular matrix proteins, such as gelatin (GL), collagen, elastin, and fibronectin, which are involved in cell adhesion, migration, proliferation, and maintenance of cell phenotype allows for a significant increase in biocompatibility of the artificially produced matrices and alters the properties of the designed materials [14–16]. It was shown that enrichment of fibers with collagen increases their tensile strength but decreases the relative elongation at break, while by contrast the addition of elastin decreases the tensile strength and increases the relative elongation at break [17]. Smooth muscle cells more efficiently attach to and proliferate on matrices made of a mixture of polyurethane (poly[4,40-methylenebis(phenyl isocyanate)-alt-1,4-butanediol/polytetrahydrofuran]) and collagen as compared with pure PU or PU-elastin blend matrices [17]. In vivo soluble tropoelastin synthesized by cells is converted into an insoluble state and strengthened by additional cross-links after its oxidation by lysyl oxidase. As a rule commercial preparations contain enzymatically hydrolyzed elastin, which is markedly different from the natural state of this protein. These preparations as well as the preparations of individual collagens are rather expensive, which considerably limits their use in tissue engineering. The most widespread collagen analog is gelatin, the product of collagen hydrolysis, which is rather inexpensive. As far as collagens are evolutionarily conserved weakly immunogenic proteins, gelatin is also virtually nonimmunogenic [18]. In addition, gelatin is known to increase cell adhesion to surfaces [15, 19, 20] and is used in manufacture of various implants [18, 21]. In particular, electrospun 3D matrices from pure gelatin are used in tissue engineering for wound healing [22].

Tecoflexes are a family of thermoplastic polyurethanes synthesized from methylenebis (cyclohexyl) diisocyanate, poly(tetramethylene glycol), and 1,4-butandyol and having a low biodegradability rate. Materials electrospun from Tecoflex EG-80A (Tec-80A) with collagen produced by coaxial electrospinning [23] or fabricated by cospraying polyurethane and gelatin [9] as well as their utility for the production of vascular implants was previously described. However, mechanical properties of the matrices made from polyurethane-gelatin (PU-GL) mixes, their behavior in aqueous media, stability, aging, and biocompatibility, and the impact of PU: GL ratio on the properties of the produced materials have not been reported yet. The properties of Tec-80A and GL suggest that their blends can be used for electrospinning of 3D matrices producing more promising materials with novel properties. Detailed description of the physicochemical and biological properties of the 3D materials, including those listed above, is necessary to expand the potential scope off use of such biomaterials for tissue engineering and regenerative medicine.

In this work, we examined the effect of gelatin content on mechanical properties and stability and structure of 3D matrices electrospun from Tec-80A-GL blend. The effects of protein fixation within fibers by glutaraldehyde (GA) on the properties and stability of matrices, as well as adhesion and proliferation of endothelial cells on the surface of these matrices, were also studied.

## 2. Materials and Methods

### 2.1. Fabrication of Electrospun Matrices

The electrospinning solutions were prepared in 1,1,1,3,3,3-hexafluoroisopropanol (HFIP) using the stock solutions of polymers (Sigma, United States): 10% PU Tec-80A (Lubrizol Advanced Materials, Europa) and 5% GL solution (Sigma, United States). Gelatin concentration in matrices is given as mass percentage of PU Tec-80A (wt/wt). The matrices with a thickness of 150–180 *μ*m were produced using an NF-103 (MESS, Japan) electrospinning device under the following conditions: the feed rate 1–1.15 ml/h, capillary-collector distance 19–20 cm, voltage 18.5–24 kV, and rotation speed of collector (diameter 3–4.5 cm) 300 rpm.

### 2.2. Analysis of Matrix Surface Structure

The microstructure of matrix surface was studied using scanning electron microscopy (SEM) as described previously [24]. Fiber diameter and pore size were calculated from SEM images according to ISO 7198:1998 [25].

To assess the stability of the fiber structure in water solutions 3D matrices were incubated in phosphate buffer (Sigma, United States) at 37°C and 5%  СО_2_ for 3, 7, 14, 21, and 28 days. After the incubation matrices were rinsed with Н_2_О, air-dried, and examined by SEM and FTIR spectroscopy.

### 2.3. Assessment of Mechanical and Physicochemical Characteristics of Matrices

Strain-stress diagrams were obtained using a universal Zwick/Roell Z100 (Germany) test bench as described in ISO 7198:1998 [25]. The electrospun matrices (thickness of 150–180 *μ*m) were carefully cut into rectangular shapes of 10 mm width and 50 mm length. Samples were placed between holders at a distance of 2–2.5 cm. Tensile testing was conducted at a rate of 10 mm×min^−1^ at room temperature (21–23°C). For the sake of statistical significance 4 specimens of each sample were tested and average values of strength and elongation at break were determined. After incubation in physiological saline solution matrices were air-dried and tensile strength of dry matrices was assessed as described.

The contact angle was determined with a Drop Shape Analyzer DSA25 (Kruss GmbH, Germany) using water as a solvent. Drop volume was set to 1 *μ*l and camera speed was 160 frames per second as recommended by manufacturer.

The porosity of matrices was calculated from the pore area and matrix surface area as (pore area/(matrix area + pore area))** ×** 100.

To assess the swelling ratio, 3D matrices were immersed in H_2_O for 2 days. Swelling ratio was calculated from the weight of wetted samples after drying with filter paper (W) and weight of the sample after complete vacuum drying (W_vd_), using the formula ((W - W_vd_)/W_vd_) × 100.

The mass loss of 3D matrices was determined after incubation in PBS at 37°C and 5%  СО_2_ for 7, 14, 21, and 28 days. The mass loss was calculated from the weight of sample before incubating (W_in_) and weight of the sample after complete vacuum drying (W_vd_) as ((W_in_ - W_vd_)/W_in_) × 100.

### 2.4. Treatment of Matrices with Glutaraldehyde

Matrices were incubated in 0.05 M NaHCO_3_ (pH 9.1) in a horizontal shaker for 20 min to moisten the material and then treated by 2% glutaraldehyde/0.05 M NaHCO_3_ solution for 2 h at room temperature. After the incubation, matrices were washed thrice with 0.05 M NaHCO_3_ (pH 9.1) for 5 min. Remaining free reactive groups were blocked by incubating the matrices in 10 mM glycine solution (pH 9.1) for 30 min followed by incubation in freshly prepared 0.1 mg/ml NaBH_4_ for 15 min. After the incubation, matrices were thoroughly washed with three changes of Н_2_О and air-dried.

### 2.5. FTIR Spectroscopy of Matrices

The infrared spectra of attenuated total reflection of matrices were recorded in the range of 4000–350 cm^–1^. For each spectrum 40 scans with a resolution of 4 cm^–1^ were collected using Cary 660 FTIR (Agilent Technologies, United States) Fourier transform infrared analyzer and a diamond GladiATR (PIKE Technologies, United States) unit.

### 2.6. Assessment of Adhesion and Viability of Endothelial Cells on the Matrix Surface

Human endothelial cells were isolated from the umbilical vein and cultivated as described previously [26, 27]. All procedures were performed in accordance with the ethical standards of the institutional committees and with the 1964 Helsinki Declaration and its later amendments or comparable ethical standards. Study was approved by ethical committees of ICBFM SB RAS and E.N. Meshalkin National Medical Research Center. Discs with a diameter of 10 mm were cut off from different matrix specimens, placed in the wells of a 48-well plate and pressed to bottom by polytetrafluoroethylene rings (outer and inner diameters, 10 and 8 mm). The discs were preincubated in culture medium for 2 h to completely moisten the matrices. Then culture medium was removed from the wells and endothelial cells were seeded into the wells (2 × 10^3^ to 20 × 10^3^ cells per well). Following 48-h cultivation the viability of endothelial cells was assessed using AlamarBlue® (Invitrogen, United States) as described previously [24]. The matrices not seeded with cells were used as control for dye sorption on the material. Seeded matrices were prepared for SEM as follows: after 48-h cultivation culture medium was removed from the wells; matrices were washed twice with phosphate buffer, fixed with 2% formaldehyde in physiological saline solution for 30 min, washed thrice with Н_2_О, and air-dried. Matrices were removed from the wells and examined by SEM as described previously [24].

## 3. Results and Discussion

### 3.1. Electrospinning and Characterization of 3D Matrices from Different Polyurethane-Gelatin Blends

The conditions for electrospinning of matrices from Tec-80A as the main polymer and GL as a supplementary protein at different weight ratios were optimized and selected parameters are shown in Table 1.

**Figure 1:**
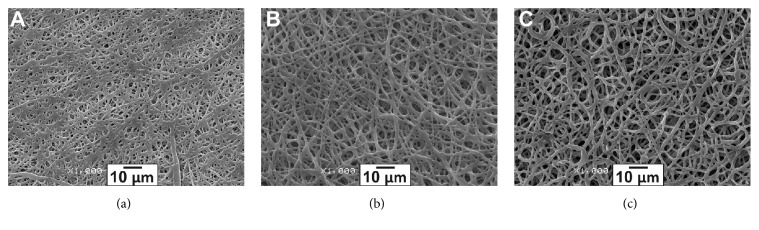
Scanning electron microscopy of matrix surface (1000x magnification). Matrices were electrospun from the following polymer solutions in hexafluoroisopropanol: (a) 3% Tec-80A + 5% GL; (b) 5% Tec-80A + 5% GL; and (c) 7% Tec-80A + 5% GL.

Microstructure of the produced matrices was examined by SEM (Figure 1). All matrices consisted of microfibers with a diameter of 0.60 ± 0.21–1.52 ± 0.40 *μ*m and pore size of 1.21 ± 0.53–7.42 ± 3.51 *μ*m, depending on the matrix composition. Higher PU concentration in the electrospinning solution yielded matrices with increased diameter of the fibers (Figure 1).

The porosity of matrices varied in the range of 10.14–47.29% depending on the concentration of PU in the electrospinning solution (Table 2). The matrices made from 5 or 7% Tec-80A with 15% GL had the highest porosity. The swelling ratio of 3D matrices was approximately 30%, independent of their composition.

Pure Tec-80A and Tec-80A–GL blend matrices demonstrated minor differences in hydrophilicity. In particular, the water contact angle values were 97.3±1.2° for pure 3% Tec-80A; 96.7±1.1° for 3% Tec-80A with 5% GL matrices; 95.5±0.8° for 3% Tec-80A with 10% GL; 95.6±0.9° for 3% Tec-80A with 15% GL; and 94.9±1.0° for 3% Tec-80A with 20% GL. Thus, the addition of GL in the electrospinning solution leads to gradual decrease of contact angle but does not make a significant contribution to the overall hydrophilicity of 3D matrices.

An increase in the protein concentration in the electrospinning blend from 5 to 20% caused shrinkage off Tec-80A matrices from 16 ± 1% to 61 ± 4.9% after moistening (5 and 7% Tec-80A matrices with proteins display a similar pattern). Note that the shrinkage factor for pure 3% Tec-80A and 3% Tec-80A with 5% GL is 17± 1% regardless of moistening.

Mechanical properties were determined for all tested materials (Table 2). The tensile strength was computed by the relation (maximum applied force)/(initial cross section); the elongation at break was calculated as ((length at break - initial length)/initial length)×100. As evident from the data in Table 2, GL concentration in electrospinning solution affects the tensile strength of the produced materials and their ultimate elongation. Low tensile strength values (4.5 ± 0.4 to 6.6 ± 0.4 MPa) are characteristic of matrices made from 7% Tec-80A as compared with 3% Tec-80A matrices (minimal value, 8.9 ± 0.6 MPa). Note that independently of Tec-80A concentration matrices with 5% GL had the lowest elongation at break, while the increase of GL concentration to 10–20% resulted in at least 50% increased elongation of the matrices. Since the matrices from 3% Tec-80A + 15% GL, 3% Tec-80A + 10% GL, and 5% Tec-80A + 15% GL displayed improved tensile strength and elasticity at break, they are expected to be a suitable material for tissue implants, since these parameters are fundamentally important for their effective performance in biological systems.

### 3.2. Assessment of In Vitro Stability of 3D Matrices

**Figure 2:**
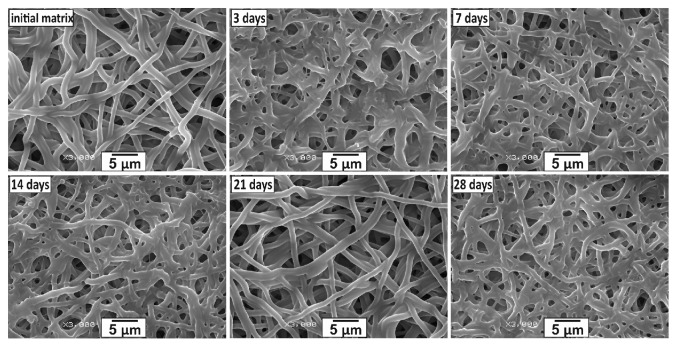
Microstructure evolution of a 5% Tec-80A + 15% GL matrix produced by electrospinning (3000x magnification).

Short and long term stability of matrices determines the range of their potential application as tissue replacement tools. Accordingly, the stability of structural and mechanical properties of the matrices was tested by incubation in phosphate buffer for 28 days at 37°C and 5%  СО_2_.  Figure 2 displays SEM images of the 5% Tec-80A + 15% GL matrix, demonstrating a change in the fiber structure and pore size after 3 and 21 days of incubation. An identical trend was observed for 3% Tec-80A + 10% GL and 3% Tec-80A + 15% GL matrices (data not shown). The data agree with other published data on the stability of matrices made from 70:30 (wt/wt) Tecophilic-GL blends [19].

**Figure 3:**
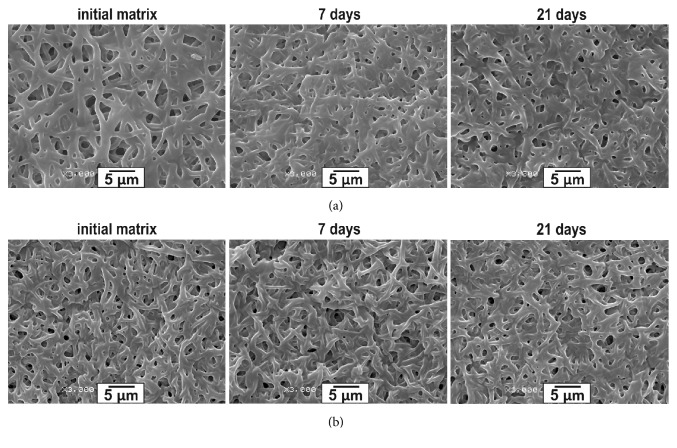
Microstructure evolution of the matrices made of 3% Tec-80A + 15% GL matrices (a) untreated and (b) treated with glutaraldehyde (3000x magnification).

In order to identify the underlying factors of the changes in fiber microstructure during the incubation in phosphate buffer, the protein contained in fibers and presumably responsible for contacts with the aqueous phase was cross-linked with glutaraldehyde. SEM examination demonstrated that GA treatment of 3% Tec-80A + 15% GL matrices (Figure 3(b)) stabilized their structure as compared with the untreated matrices (Figure 3(a)). Thus, the changes in Tec-80A- GL matrices imposed by incubation in aqueous media are associated with protein redistribution within the fibers. Note that for all tested matrices the mass loss over the course of the incubation did not exceed 1%, which was the accuracy of weighting. This is supported by our previous discovery that no more than 3% of protein is released from 3D matrices produced by electrospinning from solutions of PCL with 10% HSA (wt  :  wt) in HFIP, resulting in a total weight loss of less than 0.3%, similarly to the results of this study [24].

**Figure 4:**
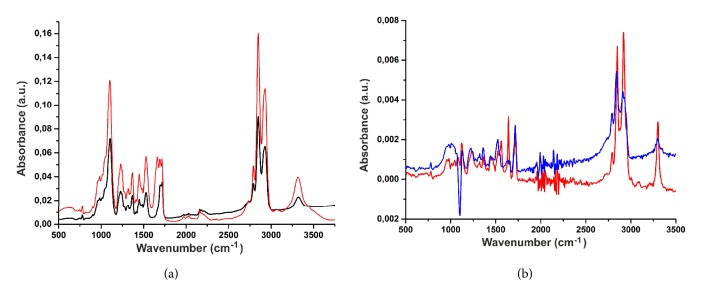
(a) FTIR spectra of pure Tec-80A matrices (black) and matrices made from Tec-80A and GL (red). (b) FTIR difference spectra of matrices made from 3% Tec-80A + 15% GL after incubation in PBS for 7 (red) or 21 (blue) days in relation to untreated matrices.

The IR spectrum (Figure 4(a)) of a matrix made of pure PU contains the absorption bands related to the valence oscillation of a bound NH group (3318 cm^–1^), valence oscillation of СН_2_ group (2950–2800 cm^–1^), valence oscillation of C=O of the free and bound urethane group (1717 and 1697 cm^–1^, respectively), amide II band (HN group bending oscillation and C–N group valence oscillation at 1527 cm^–1^), and valence oscillation of C–O–C of aliphatic ester groups in the hard and soft segments of PU (approximately 1100 cm^–1^), which complies with the published data [28–30]. The spectrum of a matrix comprising PU-GL blend has the same absorption bands as well as additional bands in the range of 1660–1640 cm^–1^, which are characteristic of the valence oscillation of C=O in the GL peptide groups [31]. Note that the spectrum of PU and GL matrices exhibits small shifts (1–5 cm^–1^) in the positions of absorption band maximums of the oscillations of NH- and C=O groups, comparative to protein-free matrices. These data suggest an interaction of polymeric protein chains and polyurethanes within the fibers.

FTIR spectroscopy also demonstrated noticeable changes in matrix structure as a result of incubation in PBS (Figure 4(b)). These changes are best detectable in the IR difference spectra, obtained by subtracting the spectrum of an untreated matrix (without incubation in PBS) from the spectrum of a matrix after the incubation for days 7 and 21. By doing so a shift in the positions of absorption band maximums in the difference spectra relative to their positions in the spectra of untreated matrices was detected (Figure 4(b)). The spectra in Figure 4(b) provide evidence that the frequency of bound C=O groups (1696 cm^–1^) in 3% Tec-80A + 15% GL matrices changes with the time of incubation in phosphate buffer, as do the oscillation frequencies of C=O groups (1660 and 1640 cm^–1^) in the protein and NH groups (3320 cm^–1^) in polyurethane. The magnitude of the observed shifts was 3–5 cm^–1^ for the oscillation of carbonyl bonds and 7–10 cm^–1^ for NH bonds. Such changes in the IR spectra suggest a change in the strength of hydrogen bonds within the system of interactions between polyurethane and protein after incubation in phosphate buffer. Additionally, small changes (1–3 cm^–1^) were observed in the oscillation frequency of CH_2_ groups (2800–2950 cm^–1^), indicating the conformational changes in methylene groups, which also suggests a change in the mutual arrangement of polyurethane and protein molecules in the fibers. It should be noted that GL, exposed at the surface of fibers, can partially dissociate from the fibers [32], which can at least partially explain the observed phenomenon of reorganizing interactions between PU and GL after the soaking of fibers in PBS.

Notably, in our conditions no hydrolysis or oxidation of PU was observed by the IR spectroscopy, judging by the lack of changes in the spectra at 1660 and 3330 cm^–1^, which appear after Tecoflex® incubation in 20–30% H_2_O_2_ for 15 days at 37°C [33]. It is likely that changes in microenvironment can influence the oxidation of polyurethane, which occurs via a mechanism involving the capture of *α*-CH_2_ hydrogen atoms adjacent to oxygen in polyester or polycarbonate polyurethanes [33]; however, this is a subject for separate study.

Thus, the absence of matrix weight loss during the incubation in water, SEM, and IR spectroscopy data suggest structural change in the PU Tec-80A-GL matrices after incubation in phosphate buffer due to the redistribution of protein in relation to polyurethane within the fibers. Presumably, the changes in the fiber structure also determine the decrease in the linear size of the materials (shrinkage) after their incubation in aqueous solutions, observed in our experiments. Indeed, it has been earlier reported that hydration of collagen matrices changes the geometry of fibers, by increasing the degree of twisting [34].

### 3.3. Changes of the Mechanical Properties of Matrices during Their Incubation in PBS

Previous studies demonstrated that, in accordance with our SEM and FTIR spectroscopy data, protein can be partially released from fibers which alters the structure of 3D matrices [24]. Since the release can influence the mechanical properties of the matrices, we examined the influence of incubation in aqueous solutions on tensile strength and elongation at break of 3D matrices (Table 3).

The data in Table 3 demonstrate that matrices not treated with glutaraldehyde in phosphate buffer had increased tensile strength after 7-day incubation. The effect gradually decreased by 21 days, but the tensile strength still remained higher than before soaking. The tensile strength of matrices with 15% GL increased by 30% after incubation in physiological saline solution versus 10% GL matrices, which demonstrated only 5–15% increased strength.

PU: GL ratio of matrices is also important: 3% Tec-80A+10% GL and 3% Tec-80A+15% GL had similar tensile strength and elongation at break, whereas 5% Tec-80A+15% GL exhibited almost 1.5 times lower strength. At that, the tensile strength of 3% Tec-80A+10% GL matrices virtually did not change during incubation in PBS, while 3% Tec-80A+15% GL and 5% Tec-80A+15% GL matrices had markedly increased strength as a result of incubation. Similarly, GA treatment had little to no effect on 3% Tec-80A+10% GL matrices but strongly affected 3% Tec-80A+15% GL matrices decreasing their tensile strength and elongation at break. Elongation at break of 3D matrices from 5% Tec-80A+15% GL increased with time and after treatment with GA. According to SEM incubation of GA-treated 3D matrices in phosphate buffer did not typically change their structure (Figure 3(b)). Evidently, the fixation of protein limits its freedom to relocate both within the fiber and at its boundary/periphery, thereby minimizing the possibility of remodeling or mutual rearrangement of molecules and formation of new intermolecular interactions during fiber hydration and loading, thus affecting the tensile strength and elasticity of the produced 3D matrices. Stability of Schiff bases and/or hydration of additional protein in the fibers during incubation in PBS can both interfere with the phenomenon observed. In any case, the increase of tensile strength and elasticity at break gained with the aging of the material can be useful in many applications, even though the basic physical chemistry dictating the mechanical behavior of protein-enriched PU matrices produced by electrospinning remains to be studied.

### 3.4. Interaction of Endothelial Cells with the Surface of PU Matrices

Chemical composition of the surface, roughness, and porosity of matrices are known to be the major factors that determine the mode of interaction of cells with matrices, namely, cell adhesion, proliferation, and migration to the inner space of tissue constructs [35, 36]. Several studies have shown that human endothelial cells prefer to attach to smooth rather than rough surfaces and more readily proliferate there [37, 38]. Note that surface roughness within the 10–135 nm range has only marginal effect on the cell ability to attach to the matrix surface, while roughness exceeding 287 nm inhibits cell attachment [37, 38]. According to other data, the change in fiber diameter in the range of 0.3 to 1.3 *μ*m for the matrices electrospun from polycaprolactone has no effect on cell adhesion and proliferation and does not affect the migration of fibroblasts into the scaffolds [39]. The matrices with fibers of large diameter behave quite differently: a change in the diameter of glass wires from 1 to 85 *μ*m altered the migration of epithelial cells. When wire diameter was less than 40 *μ*m, epithelial cells formed circular arrangements around the fibers, which enhanced their collective migration [40], whereas for wire diameter over 40 *μ*m the cells assumed morphology characteristic of flat surfaces and collective migration was absent. Thus, the ability of cells to wrap around the fibers is a key factor for their effective migration in fibrous materials.

Another factor that determines the colonization of the inner space of 3D matrices by defining the mechanism of cell-matrix interaction is the pore size [41]. Depending on the cell type pores of at least 5–12 *μ*m are required for efficient colonization [42]. Moreover, pore size has a greater effect on the proliferation of dermal fibroblasts in polycaprolactone matrices than the diameter of the fibers [43]. According to fluorescence microscopy, fibroblasts successfully proliferated and colonized matrices with 6 *μ*m pores, but an increase of the pore diameter from 12 to 23 *μ*m resulted in altered cell morphology and patterns of cell arrangement around/along individual fibers, interfering with the efficient colonization of matrices. The matrices produced from 3% Tec-80A + 15% GL, 3% Tec-80A + 10% GL, and 5% Tec-80A + 15% GL had fiber diameter in the range of 0.6–1.52 *μ*m and pore size in the range of 1.21–7.42 *μ*m (Table 2), thus suggesting that their surface should be efficiently colonized by cells. However, due to the high elasticity of matrices, the shape and pore size of the matrices can change even at low levels of mechanical stress, allowing for cell migration into the matrices that were subjected to mechanical deformations.

**Figure 5:**
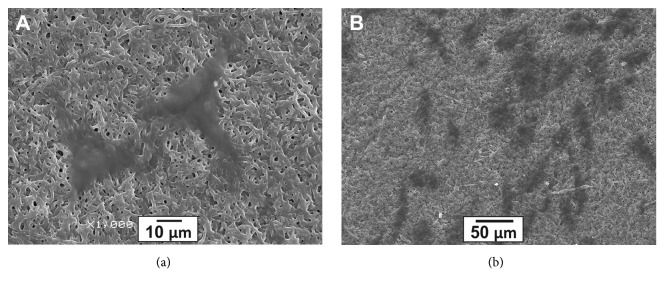
Endothelial cells attached to surface of 3D matrices after 48 h cultivation. SEM images ((a) 1000**×** magnification; (b) 300× magnification).

Earlier studies have demonstrated that endothelial cells efficiently interact with the surface of electrospun matrices produced from collagen, chitosan, and PU blends (60* *:* *15* *:* *25) [44]. Endothelium formed on the inner surface of vascular implants plays an important role in its functioning [45]. In order to assess the potential of Tec-80A-GL matrices for use in vascular implants, the interaction of human endothelial cells with the matrices was examined by SEM (Figure 5). It is evident that endothelial cells attached to the surface of matrices retain their typical morphology, which is characteristic of endothelial cells growing on cell culture plastic.

**Figure 6:**
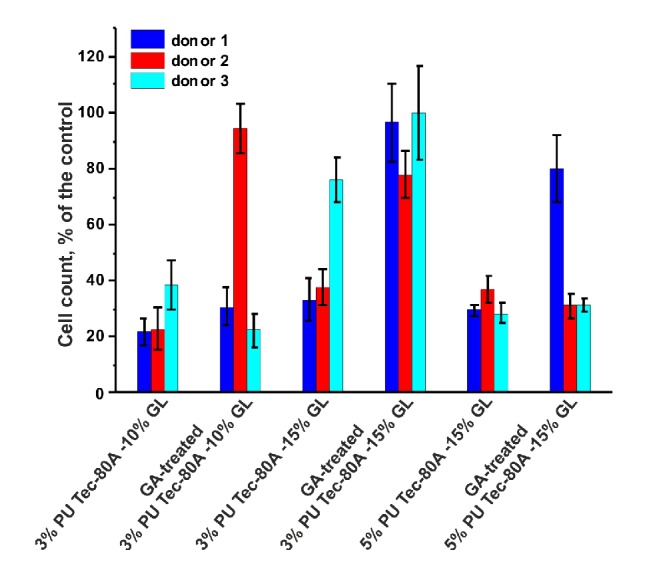
Viability of endothelial cells on the surface of different matrices after 48-h cultivation (mean of three replicates with standard deviation).

The amount of viable cells on the surface of matrices was assessed after 48 h of cultivation using a commercial reagent, AlamarBlue. It is known that the cells from different biological donors differ in their ability to proliferate on cell culture plastic [46]. As such cells of three biological donors were used in our experiments.  Figure 6 illustrates the viability of endothelial cells on the surface of different matrices. The data verified that endothelial cells from different donors do indeed differ in their ability to proliferate on the surface of the same matrix. An increase in the GL concentration improves the adhesion and proliferation of cells on the surface of the matrices, as demonstrated by 3% Tec-80A + 15% GL as compared with 3% Tec-80A + 10% GL. Glutaraldehyde treatment also promoted cell growth, most likely due to its stabilizing effect on fiber structure after hydration. Our data suggests that GA-treated matrices made from 3% Tec-80A + 15% GL are most suited for cell growth.

Thus, the results of this study demonstrate that GL concentration in the electrospinning blend determines the mechanical properties of the produced matrices, namely, their tensile strength and elongation, which agrees with the data on the matrices from 3% polycaprolactone with 10–30% GL [15]. In addition, SEM and FTIR spectroscopy both indicate that changes in the structure of matrices after incubation in phosphate buffer are caused by hydration and occur due to the redistribution of protein within the fibers relative to polyurethane. Glutaraldehyde treatment fixes the protein within the fibers, thereby making the surface protein more stable and decreasing protein solubility, i.e., protein assumes a state resembling the biological surface of insoluble collagens and/or elastins, assembled from soluble precursors. The surface of GA-treated 3% Tec-80A + 15% GL matrices was most suitable for binding of primary human endothelial cells independently of the cell donor.

## 4. Conclusions

Thus, the obtained data on mechanical properties of Tec-80A-GL blended matrices, their behavior in aqueous media, stability and changes in their mechanical properties in aqueous media, and biocompatibility demonstrate their high potential as materials for use in engineering of elastic tissues like vascular grafts, valves, patches for blood vessels and bowels. One important implication of this study is that it is necessary to keep in mind that moistening can alter the properties of blended matrices and not only cause their shrinkage but also change the mechanical properties and alter the aging of the materials. Introduction of protein in the fibers and its fixation are a valid approach to influence the stability and mechanical and biological properties of the resulting matrices.

## Figures and Tables

**Table 1:** Electrospinning conditions for producing matrices from Tec-80A-GL blends.

Blend composition	Electrospinning parameters		
	Voltage, kV	Solution supply rate, ml/h	Distance between spinneret and collector, cm
3% Tec-80A + 5% GL	19.0	1.3	20
3% Tec-80A + 10% GL	18.5	1.3	20
3% Tec-80A + 15% GL	18.5	1.2	19.5
3% Tec-80A + 20% GL	20.0	1.2	19.5
5% Tec-80A + 5% GL	19.5	1.3	20
5% Tec-80A + 10% GL	22.5	1.4	20
5% Tec-80A + 15% GL	21.0	1.4	18.5
5% Tec-80A + 20% GL	20.0	1.3	18.5
7% Tec-80A + 5% GL	21.0	1.5	20
7% Tec-80A + 10% GL	22.1	1.3	20
7% Tec-80A + 15% GL	24.0	1.2	19
7% Tec-80A + 20% GL	24.0	1.0	19

**Table 2:** Structural characteristics and mechanical properties of 3D matrices.

Matrix composition	Structural characteristics			Mechanical properties	
	Fiber diameter, *µ*m	Pore size, *µ*m	Porosity, %	Tensile strength, MPa	Elongation at break, %
3% Tec-80A + 5% GL	0.73 ± 0.26	1.52 ± 0.67	11.78	8.9 ± 0.6	238 ± 39
3% Tec-80A + 10% GL	0.60 ± 0.21	1.24 ± 0.44	10.38	14.9 ± 1.6	398 ± 20
3% Tec-80A + 15% GL	0.66 ± 0.24	1.21 ± 0.53	10.14	15.6 ± 0.8	392 ± 51
3% Tec-80A + 20% GL	0.64 ± 0.22	1.33 ± 0.91	11.76	9.8 ± 0.5	319 ± 37
5% Tec-80A + 5% GL	1.31 ± 0.55	3.07 ± 1.62	27.68	6.9 ± 1.1	261 ± 56
5% Tec-80A + 10% GL	1.30 ± 0.29	2.81 ± 1.38	21.73	7.5 ± 0.4	318 ± 63
5% Tec-80A + 15% GL	1.52 ± 0.40	7.42 ± 3.51	38.36	10.8 ± 0.3	376 ± 54
5% Tec-80A + 20% GL	1.30 ± 0.42	4.31 ± 1.89	32.88	3.4 ± 0.1	393 ± 67
7% Tec-80A + 5% GL	1.15 ± 0.28	4.81 ± 2.64	39.71	4.5 ± 0.4	260 ± 12
7% Tec-80A + 10% GL	1.28 ± 0.49	5.13 ± 2.45	39.80	5.5 ± 0.4	323 ± 67
7% Tec-80A + 15% GL	1.20 ± 0.38	6.42 ± 4.53	47.29	6.6 ± 0.4	357 ± 61
7% Tec-80A + 20% GL	1.32 ± 0.64	3.67 ± 1.42	31.35	4.6 ± 0.9	310 ± 38

**Table 3:** Mechanical characteristics of untreated and GA-treated matrices after incubation in PBS.

Matrix		Mechanical characteristics of matrix*∗*	Incubation in phosphate buffer for		
			Control	7 days	21 days
**Untreated**	3% Tec-80A + 10% GL	Tensile strength	14.9 ± 1.6	15.7 ± 0.5	16.8 ± 0.9
		Elongation at break	398 ± 20	296 ± 38	298 ± 25
	3% Tec-80A + 15% GL	Tensile strength	15.6 ± 0.8	20.4 ± 1.7	17.0 ± 1.2
		Elongation at break	392 ± 51	293 ± 24	383 ± 47
	5% Tec-80A + 15% GL	Tensile strength	10.8 ± 0.3	14.2 ± 1.3	13.8 ± 1.2
		Elongation at break	376 ± 54	379 ± 37	527 ± 42

**GA-treated **	3% Tec-80A + 10% GL	Tensile strength	15.1 ± 1.8	17.3 ± 2.8	18.1 ± 0.8
		Elongation at break	298 ± 31	323 ± 24	304 ± 41
	3% Tec-80A + 15% GL	Tensile strength	9.2 ± 0.5	7.7 ± 1.2	9.9 ± 0.8
		Elongation at break	346 ± 27	477 ± 39	472 ± 64
	5% Tec-80A + 15% GL	Tensile strength	9.5 ± 1.6	11.0 ± 2.1	12.0 ± 1.1
		Elongation at break	287 ± 39	343 ± 51	530 ± 67

*∗*: tensile strength, MPa; elongation at break, %.

## Data Availability

The datasets generated and analyzed during the current study are available from the corresponding author on reasonable request.
